# PET/CT radiomics–biomarker diagnostic model for identifying lymphoma in fever of unknown origin

**DOI:** 10.3389/fimmu.2026.1797162

**Published:** 2026-04-21

**Authors:** Fenglian Jing, Xinchao Zhang, Ranliang Hua, Jingjie Zhang, Kang Li, Xiaoyu Cai, Congna Tian, Qiang Wei, Yanzhu Bian

**Affiliations:** 1 Department of Nuclear Medicine, The Fourth Hospital of Hebei Medical University, Shijiazhuang, Hebei, China; 2 Department of Nuclear Medicine, Hebei General Hospital, Shijiazhuang, Hebei, China; 3 Department of Emergency, Hebei General Hospital, Shijiazhuang, Hebei, China

**Keywords:** ^18^F-FDG PET/CT, fever of unknown origin, lymph node diseases, lymphoma, procalcitonin, serum amyloid A

## Abstract

**Background:**

Fever of unknown origin (FUO) with lymphadenopathy poses a substantial diagnostic challenge, particularly in differentiating malignant lymphoma from benign lymph node disorders. Although PET/CT plays a crucial role in lesion detection, inflammatory and immune-mediated lymphadenopathies often exhibit metabolic patterns overlapping with lymphoma, leading to limited diagnostic specificity. Radiomics enables high-throughput extraction of quantitative imaging features that capture intratumoral heterogeneity beyond visual assessment, while blood-based biomarkers reflect systemic inflammatory and metabolic states. This study aimed to develop and validate a multimodal diagnostic model integrating PET/CT radiomics and clinical biomarkers to improve the discrimination of lymphoma from benign lymphadenopathy in patients with FUO.

**Methods:**

This retrospective study included FUO who underwent PET/CT. Lymph nodes were selected if the short-axis diameter was ≥1 cm on CT or if metabolic activity exceeded the mediastinal blood pool. Clinical and laboratory data were collected, and radiomics features were extracted from 40%SUVmax–based VOIs using LIFEx. Patients were randomly divided into training and testing sets (7:3). A biomarker model, radiomics model, and combined model were constructed using logistic regression, with feature selection performed via the least absolute shrinkage and selection operator (LASSO) for radiomics. Model performance was evaluated using receiver operating characteristic (ROC) curves, and DeLong’s test. A nomogram was developed for individualized prediction.

**Results:**

A total of 203 patients were enrolled (114 lymphoma, 89 benign). In the training cohort, under the curve (AUC)s for the biomarker, radiomics, and combined models were 0.924, 0.903, and 0.970, respectively, with the combined model showing significantly superior performance. In the testing cohort, the corresponding AUCs were 0.903, 0.912, and 0.958, again demonstrating the highest accuracy for the combined model. Key predictors in the final model included procalcitonin (PCT), serum amyloid A (SAA), and PET/CT radiomics features. A nomogram was generated to facilitate individualized risk estimation.

**Conclusion:**

PET/CT radiomics provides strong discriminatory ability for differentiating lymphoma from benign lymphadenopathy in FUO. Incorporating multidimensional biomarkers such as PCT and SAA further enhances diagnostic performance.

## 1 Introduction

Fever of unknown origin (FUO) is defined as a persistent temperature of ≥38.3 °C for more than three weeks without an identified cause, despite three days of inpatient or outpatient evaluation (1, 2). As a complex clinical entity, FUO encompasses a broad etiological spectrum that includes infections, malignancies, non-infectious inflammatory disorders, and other miscellaneous conditions (3, 4). Lymphadenopathy is frequently observed in FUO patients (5, 6), which markedly increases diagnostic complexity, as physicians must differentiate malignant lymphoma from a variety of benign lymph node disorders. This challenge is further compounded by the significant overlap in clinical manifestations and conventional imaging features between lymphoma and certain benign conditions—such as adult-onset Still’s disease and necrotizing lymphadenitis—which often present with high fever, rash, arthralgia, and hypermetabolic lymph nodes on PET/CT, thereby limiting the diagnostic accuracy of traditional modalities (7, 8).

While FDG PET/CT is indispensable for identifying hypermetabolic lesions, its interpretation in FUO is uniquely challenging. In this setting, inflammatory and immune-mediated lymphadenopathies frequently demonstrate metabolic activity comparable to that of malignant lymphoma, substantially compromising diagnostic specificity (9–12). In recent years, radiomics has emerged as a promising approach to address these limitations. By extracting high-dimensional quantitative features from PET/CT images, radiomics captures subtle heterogeneity and texture patterns that are undetectable to the naked eye (13). Machine-learning-based radiomics models—particularly those using random forest algorithms—have demonstrated excellent performance across multiple tumor types (14–19). In FUO with lymphadenopathy, a PET/CT-based radiomics random forest model outperformed support vector machines, logistic regression, and k-nearest neighbor algorithms in both training and testing datasets, highlighting its substantial diagnostic potential (20).

In addition, blood-based biomarkers—reflecting systemic pathophysiological states—play indispensable roles in FUO etiological assessment. Biomarkers related to inflammation and nutritional status, such as procalcitonin (PCT), ferritin (FER), serum amyloid A (SAA), and albumin (ALB), have been shown to correlate strongly with differential diagnosis between lymphoma and benign lymphadenopathy (21, 22). These biomarkers provide macroscopic insights into disease biology and offer advantages of clinical accessibility and interpretability.

Given the inherent limitations of single-modality diagnostics, multimodal integration has become an emerging trend to improve diagnostic accuracy in FUO with lymphadenopathy. Radiomics quantifies spatial structural and metabolic heterogeneity, while blood biomarkers reflect systemic molecular alterations. Their combination enables complementary information acquisition, supporting more comprehensive disease characterization. Such integration may offer more reliable guidance in complex clinical settings.

Based on these advancements and clinical needs, the present study aimed to construct and validate a multimodal diagnostic model integrating PET/CT radiomics and biomarkers to distinguish lymphoma from benign lymphadenopathy in FUO patients. By leveraging complementary information from both modalities, we sought to develop a diagnostic tool with high accuracy and strong interpretability, providing clinicians with objective and quantitative decision support. Ultimately, this may optimize diagnostic strategies, reduce unnecessary invasive procedures, and improve patient outcomes.

## 2 Materials and methods

### 2.1 Study population

This retrospective study included FUO patients who underwent PET/CT examination at our institution from January 2018 to December 2023. FUO was diagnosed according to the criteria described in the Introduction. Inclusion criteria were as follows: (1) confirmed FUO with ^18^F-FDG PET/CT performed; (2) complete clinical and laboratory data; (3) PET/CT indicating lymphadenopathy with either metabolic activity exceeding that of the mediastinal blood pool or a short-axis diameter ≥1 cm on axial CT; (4) definitive diagnosis—benign diseases confirmed via clinical follow-up or biopsy, and malignant cases confirmed by pathology. Exclusion criteria: (1) incomplete clinical or laboratory data; (2) immunodeficiency-related or nosocomial FUO; (3) presence of non-lymphoma solid tumors or history of malignancy. Ethical approval was obtained from the institutional review board (Approval No. 2022KY288 and No. 2024-LW-081).

Clinical and laboratory parameters were collected, including: gender, age, hyperpyrexia, joint pain, rash, weight loss, hepatomegaly, splenomegaly, blood analysis (BA), blood cultures (BC), total protein (TP), ALB, lactate dehydrogenase (LDH), creatine kinase (CK), C-reactive protein (CRP), erythrocyte sedimentation rate (ESR), PCT, FER, SAA, alanine aminotransferase (ALT), aspartate aminotransferase (AST), antinuclear antibodies (ANA), rheumatoid factor (RF), immunofixation electrophoresis (IFE), and interferon-gamma release assay (IGRA).

### 2.2 ^18^F-FDG PET/CT examination

All examinations were performed on a GE Discovery Elite PET/CT scanner using ^18^F-FDG with radiochemical purity ≥90%. Patients fasted for ≥6 hours before imaging, and blood glucose levels were maintained ≤11.1 mmol/L. ^18^F-FDG (3.70-5.55 MBq/kg) was intravenously administered, followed by a 60-minute uptake period. Scanning was performed from the skull base to mid-thigh in 6–7 bed positions (2.5 min per bed) using 3D time-of-flight (TOF) mode. Low-dose CT (120 kV, 100 mA, 3.3-mm slice thickness) was used for attenuation correction. Oral contrast (600 mL diatrizoate solution) was administered for gastrointestinal delineation. Fused PET/CT images were subsequently reconstructed.

### 2.3 Image segmentation and metabolic parameter extraction

Two nuclear medicine physicians (>5 years of experience) manually delineated lymph nodes using LIFEx software (version 7.3.0). Lymph nodes with short-axis ≥1 cm or metabolism greater than the mediastinal blood pool were segmented to obtain volumes of interest (VOIs). VOIs were refined using a 40% maximum standardized uptake value (SUVmax) threshold. Conventional metabolic parameters, including SUVmax, SUVmean, total metabolic tumor volume (TMTV), and total lesion glycolysis (TLG), were extracted.

**Figure 1:**
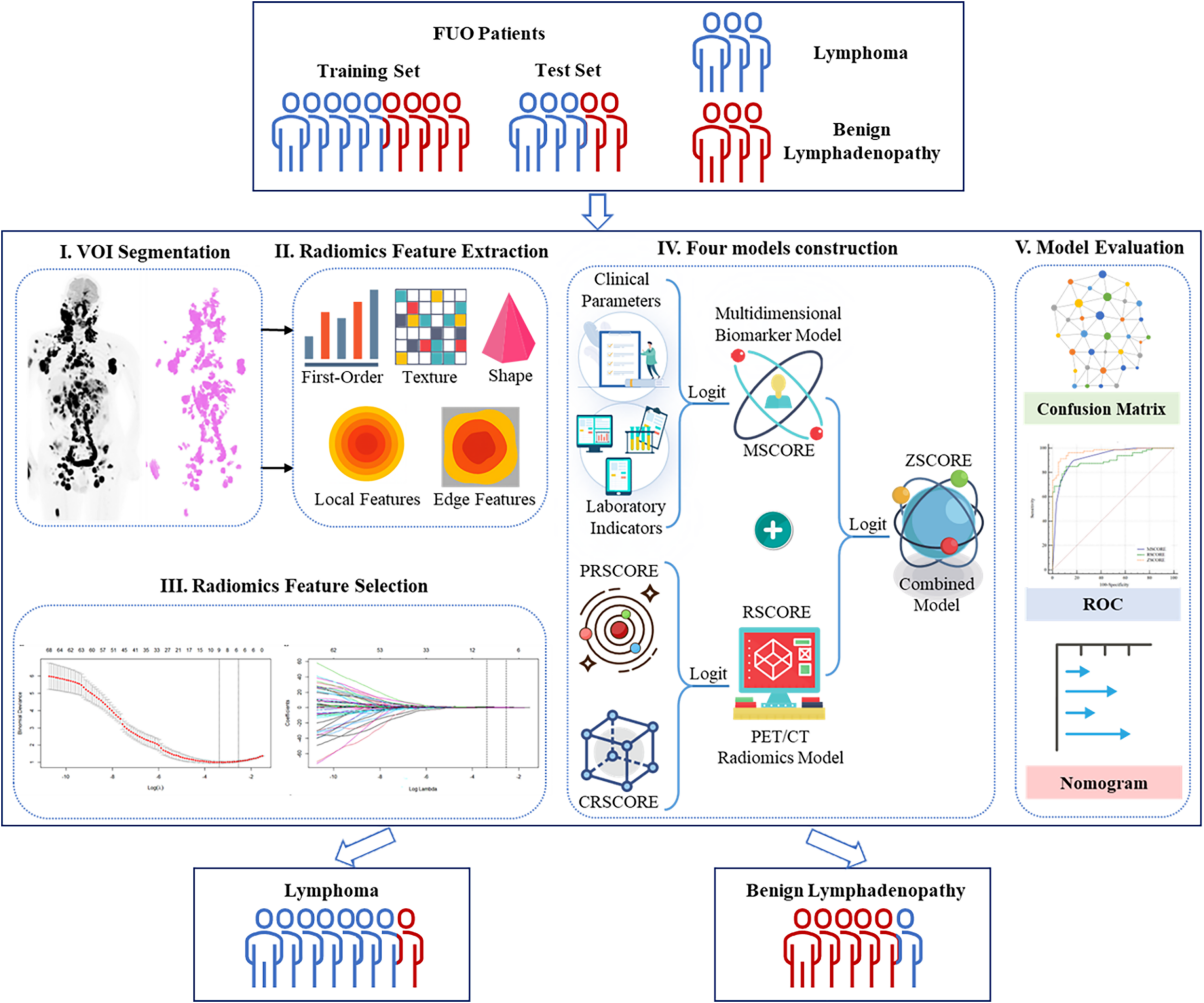
Workflow of the study.

Given the accurate registration between PET and CT, radiomics features from both modalities were extracted from the same VOIs for subsequent modeling. The schematic workflow is shown in **Figure 1**.

### 2.4 Model construction

Cases were randomly divided into training and testing sets at a 7:3 ratio.

#### 2.4.1 Multidimensional biomarker model

Univariate and multivariate logistic regression analyses were performed to identify independent biomarkers associated with the differentiation of lymphoma from benign lymphadenopathy (*P* < 0.05). The retained predictors were used to establish a biomarker-based scoring system (MSCORE).

#### 2.4.2 Radiomics model

All radiomic features underwent standardization prior to selection. The least absolute shrinkage and selection operator (LASSO) with tenfold cross-validation was applied to identify non-zero-coefficient features from PET- and CT-derived radiomics. Separate PET and CT radiomics scores (PRSCORE and CRSCORE) were calculated and subsequently integrated through logistic regression to generate the overall radiomics score (RSCORE).

#### 2.4.3 Combined model

To leverage complementary information, the biomarker model and radiomics model were further integrated using multivariate logistic regression. This yielded the combined prediction model and its corresponding composite score (ZSCORE) for estimating the probability of lymphoma.

### 2.5 Model evaluation and validation

In both training and test sets, diagnostic performance for differentiating lymphoma from benign lymphadenopathy was assessed using receiver operating characteristic (ROC) curves and area under the curve (AUC). DeLong’s test (MedCalc 19.6.4) was used to compare AUCs between models. A nomogram incorporating key predictors was constructed to visualize individualized risk estimation.

### 2.6 Statistical analysis

Statistical analyses were performed using SPSS 26.0 and R 4.2.2. Continuous variables conforming to normal distribution were expressed as $\bar{x}$ ± s and compared using independent t-tests; non-normally distributed variables were presented as *M* (Q1, Q3) and compared using Mann–Whitney *U* tests. Categorical variables were compared with chi-square or Fisher’s exact tests. A two-sided *P* < 0.05 was considered statistically significant.

## 3 Results

### 3.1 Patient characteristics

A total of 325 FUO patients with lymphadenopathy were initially screened. Twenty-five were excluded due to concurrent solid tumors, seven for prior malignancy, 89 for undetermined diagnoses at follow-up, and one for incomplete data. Ultimately, 203 patients were included: 114 with lymphoma and 89 with benign lymphadenopathy. The cohort included 99 males and 104 females, with a mean age of 48.5 ± 20.9 years (range: 2–85 years). Patients were randomly assigned into training (n=142) and test (n=61) set, with a comparable distribution of disease categories between the two cohorts (training set: 80 lymphoma and 62 benign cases; test set: 34 lymphoma and 27 benign cases).

### 3.2 Model construction

Univariate and multivariate logistic regression analyses of multidimensional biomarkers identified hyperpyrexia (temperature >39 °C=1), rash, PCT, and SAA as independent predictors for distinguishing lymphoma from benign lymphadenopathy (all *P* < 0.05) (**Table 1**).

**Table 1:** Univariate and multivariate logistic regression analyses of multidimensional biomarkers.

Characteristics	Univariate analysis		Multivariate analysis	
	OR (95% CI)	*P*	OR (95% CI)	*P*
Age	1.008 (0.993-1.024)	0.286	–	–
Gender	0.467 (0.237-0.918)	0.027	0.603 (0.191-1.903)	0.388
Hyperpyrexia*	0.106 (0.037-0.299)	<0.001	0.135 (0.025-0.73)	0.020
Joint Pain	0.043 (0.01-0.194)	<0.001	0.541 (0.086-3.385)	0.511
Rash	0.047 (0.01-0.208)	<0.001	0.06 (0.006-0.591)	0.016
Weight Loss	1.838 (0.538-6.275)	0.331	–	–
Hepatomegaly	1.595 (0.383-6.647)	0.522	–	–
Splenomegaly	1.253 (0.642-2.445)	0.509	–	–
WBC	0.502 (0.275-0.917)	0.025	0.765 (0.199-2.936)	0.696
LYM	1.153 (0.542-2.455)	0.712	–	–
NEU	0.275 (0.13-0.582)	<0.001	1.073 (0.187-6.151)	0.937
RBC	0.891 (0.462-1.72)	0.731	–	–
Hb	0.899 (0.503-1.607)	0.720	–	–
PLT	0.633 (0.275-1.458)	0.282	–	–
BC	0.249 (0.025-2.454)	0.234	–	–
TP	0.654 (0.312-1.372)	0.261	–	–
ALB	0.291 (0.145-0.586)	<0.001	1.853 (0.42-8.18)	0.415
LDH	0.867 (0.453-1.661)	0.667	–	–
CK	2.222 (1.126-4.387)	0.021	1.916 (0.588-6.245)	0.281
CRP	0.134 (0.054-0.329)	<0.001	0.458 (0.075-2.802)	0.398
ESR	0.142 (0.063-0.319)	<0.001	1.431 (0.296-6.926)	0.656
PCT	0.046 (0.019-0.111)	<0.001	0.09 (0.024-0.346)	<0.001
FER	0.25 (0.1-0.622)	0.003	1.635 (0.291-9.172)	0.576
SAA	0.082 (0.036-0.185)	<0.001	0.17 (0.037-0.791)	0.024
ALT	0.234 (0.091-0.606)	0.003	0.819 (0.097-6.894)	0.854
AST	0.211 (0.092-0.487)	<0.001	0.876 (0.11-6.992)	0.901
ANA	0.309 (0.101-0.943)	0.039	0.34 (0.061-1.904)	0.220
RF	0.444 (0.102-1.935)	0.280	–	–
IFE	3.211 (0.35-29.475)	0.302	–	–
IGRA	1.093 (0.33-3.625)	0.884	–	–

OR, Odds Ratio; WBC, White Blood Cell; LYM, Lymphocyte; NEU, Neutrophil; RBC, Red Blood Cell; Hb, Hemoglobin; PLT, Platelet; BC, Blood Cultures; TP, Total Protein; ALB, Albumin; LDH, Lactate Dehydrogenase; CK, Creatine Kinase; CRP, C-Reactive Protein; ESR, Erythrocyte Sedimentation Rate; PCT, Procalcitonin; FER, Ferritin; SAA, Serum Amyloid A; ALT, Alanine Aminotransferase; AST, Aspartate Aminotransferase; ANA, Antinuclear Antibodies; RF, Rheumatoid Factor; IFE, Immunofixation Electrophoresis; IGRA, Interferon Gamma Release Assay.

*Hyperpyrexia refers to a body temperature greater than 39°C.

The biomarker-based score was calculated as:


MSCORE=3.272−2.000×Hyperpyrexia (temperature>39°C=1)−2.816×Rash (presence of rash=1)−2.403×PCT (PCT≥0.06ng/mL=1)−1.771×SAA (SAA≥10mg/L=1)

**Figure 2:**
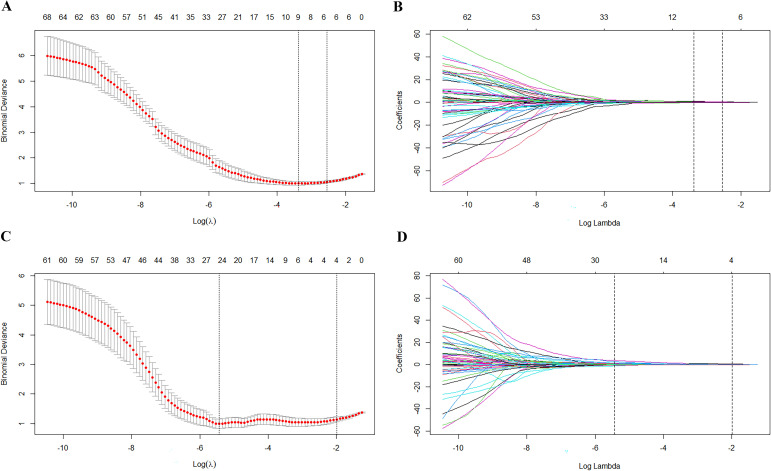
Optimal radiomic features identified using the LASSO algorithm. The tuned parameters (Lambda) and the distribution of LASSO coefficients for the LASSO model with the smallest criterion were determined for predicting lymphoma and benign lymphadenopathy through a 10-fold cross-validation. The vertical dashed line represents the optimal value obtained using the minimum criterion. **(A, B)** PET features; **(C, D)** CT features.

A total of 124 PET-based and 123 CT-based radiomics features were extracted using LIFEx software. After feature standardization and dimensionality reduction with LASSO regression, 6 PET radiomics features and 4 CT radiomics features were retained. The LASSO coefficient plots for PET and CT features are shown in **Figure 2**, and the selected features with their corresponding coefficients are summarized in **Table 2**.

**Table 2:** LASSO analysis results of PET and CT radiomic features.

Feature label	Feature name	Coefficient
P1	MORPHOLOGICAL_SurfaceToVolumeRatio	-0.1470
P2	MORPHOLOGICAL_Maximum3DDiameter	0.5373
P3	INTENSITY-BASED_Skewness	-0.1392
P4	INTENSITY-HISTOGRAM_IntensityHistogramMode	0.4152
P5	INTENSITY-HISTOGRAM_RootMeanSquare	-0.1717
P6	NGTDM_Coarseness	-0.0153
C1	MORPHOLOGICAL_SurfaceToVolumeRatio	-0.0346
C2	MORPHOLOGICAL_Maximum3DDiameter	0.0245
C3	GLRLM_LongRunHighGreyLevelEmphasis	0.5028
C4	GLSZM_ZonePercentage	-0.3316

Radiomics scores were calculated using the following formulas:


PRSCORE=0.4338−0.1470×P1+0.5373×P2−0.1392×P3+0.4152×P4−0.1717×P5−0.0153×P6

CRSCORE=0.3393−0.0346×C1+0.0245×C2+0.5028×C3−0.3316×C4

Univariate and multivariate logistic regression analyses demonstrated that both PRSCORE and CRSCORE were independent predictors for differentiating lymphoma from benign lymphadenopathy (all *P* < 0.05) (**Table 3**).

**Table 3:** Univariate and multivariate logistic regression analyses of PET/CT radiomic features.

Characteristics	Univariate analysis		Multivariate analysis	
	OR (95% CI)	*P*	OR (95% CI)	*P*
PRSCORE	13.471 (5.6-32.407)	<0.001	5.271 (2.01-13.824)	<0.001
CRSCORE	19.588 (7.395-51.883)	<0.001	6.99 (2.334-20.936)	<0.001

The overall PET/CT radiomics score was calculated as:


RSCORE=−0.3558+1.6623×PRSCORE+1.9445×CRSCORE

The independent predictors from the multidimensional biomarker model were then combined with the radiomics score to establish the final multimodal model. Multivariate logistic regression demonstrated that PCT, SAA, and RSCORE were independent predictors (all *P* < 0.05) (**Table 4**). The combined model score was defined as:

**Table 4:** Univariate and multivariate logistic regression analyses of the combined model.

Characteristics	Univariate analysis		Multivariate analysis	
	OR (95% CI)	*P*	OR (95% CI)	*P*
Hyperpyrexia	0.106 (0.037-0.299)	<0.001	0.239 (0.042-1.356)	0.106
Rash	0.047 (0.01-0.208)	<0.001	0.198 (0.025-1.565)	0.125
PCT	0.046 (0.019-0.111)	<0.001	0.062 (0.015-0.261)	<0.001
SAA	0.082 (0.036-0.185)	<0.001	0.18 (0.046-0.713)	0.015
RSCORE	2.718 (1.946-3.796)	<0.001	2.518 (1.609-3.939)	<0.001


ZSCORE=2.9041−2.7848×PCT−1.7137×SAA+0.9233×RSCORE

### 3.3 Model evaluation and validation

**Figure 3:**
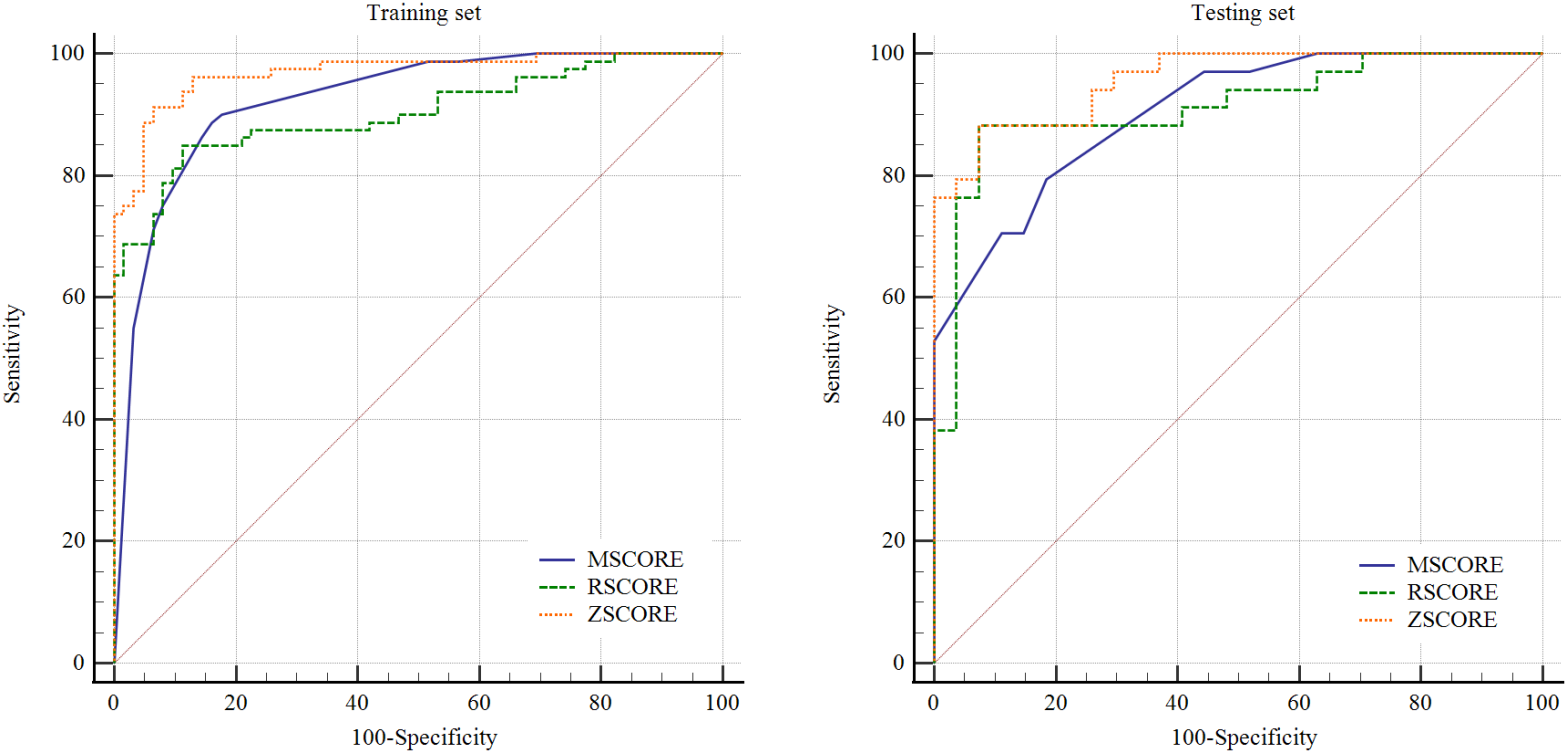
Receiver operating characteristic (ROC) curves of the multidimensional biomarker model, PET/CT radiomic model, and combined model for distinguishing lymphoma from benign lymphadenopathy.

In the training cohort, the multidimensional biomarker model, radiomics model, and composite model demonstrated strong discriminatory performance, with AUCs of 0.924 (95%CI: 0.880-0.968), 0.903 (95%CI: 0.853-0.953), and 0.970 (95%CI: 0.945-0.994), respectively. DeLong’s test indicated that the composite model significantly outperformed both the biomarker and radiomics models (*P* = 0.014 and 0.002; **Figure 3**). In the testing cohort, the AUCs of the three models were 0.903 (95%CI: 0.834-0.972), 0.912 (95%CI: 0.836-0.988), and 0.958 (95%CI: 0.916-0.999), respectively. Although the composite model again showed numerically superior performance, the differences compared with the individual models were not statistically significant (*P* = 0.122 and 0.089; **Figure 3**).

**Figure 4:**
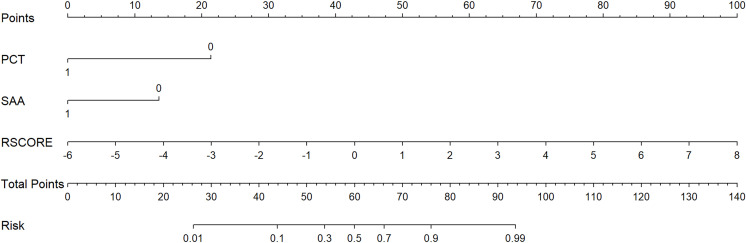
Nomogram based on the combined model.

Given the excellent performance of the final multimodal model incorporating PCT, SAA, and PET/CT radiomics features in distinguishing lymphoma from benign lymphadenopathy, we further developed a personalized nomogram to visually present the contribution of each predictor and to facilitate individualized probability estimation (**Figure 4**).

**Figure 5:**
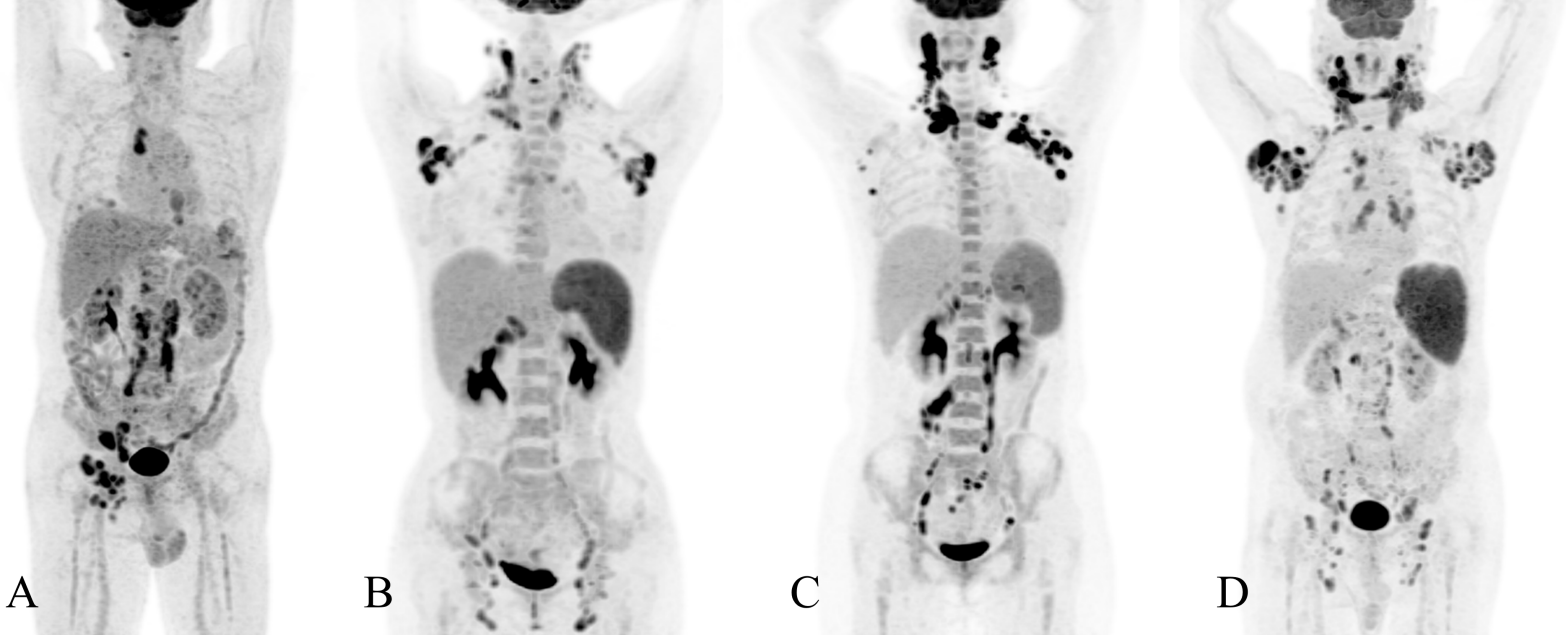
Representative cases illustrating the clinical application of the nomogram-based diagnostic model. **(A)** Adult-onset Still’s disease; **(B)** Necrotizing lymphadenitis; **(C)** Follicular T-cell lymphoma; **(D)** Diffuse large B-cell lymphoma. In all four cases, both procalcitonin (PCT) and serum amyloid A (SAA) were positive. Details of how to implement the regression formula for probability estimation, *P* = 1/(1+*e^-ZSCORE^*), are provided in the Data **Supplementary Data Sheet 1**. The predicted malignancy probabilities were 9.56% for adult-onset Still’s disease, 45.35% for necrotizing lymphadenitis, 65.25% for follicular T-cell lymphoma, and 75.29% for diffuse large B-cell lymphoma, demonstrating a stepwise increase in malignant risk that is highly consistent with the underlying pathological diagnoses.

To further illustrate the clinical applicability and interpretability of the nomogram-based diagnostic model, four representative cases with distinct pathological diagnoses are presented in **Figure 5**. Despite similar inflammatory biomarker profiles, the model yielded substantially different malignancy probabilities across the three cases.

## 4 Discussion

FUO represents a diagnostically challenging syndrome spanning infectious, malignant, and inflammatory etiologies, often requiring extensive clinical expertise. Traditional diagnostic methods—including medical history, physical examination, laboratory testing, and basic imaging—are frequently insufficient in complex cases. Lymphadenopathy is common in FUO (23, 24), and lymphoma is the predominant malignant cause, accounting for 56.2% of cases (114/203) in our cohort. Conversely, infections, adult-onset Still’s disease, and necrotizing lymphadenitis constitute the main benign etiologies (6, 9, 20). These benign conditions share substantial overlap with lymphoma in clinical manifestations, imaging characteristics, and laboratory profiles, exacerbating diagnostic uncertainty.

Adult-onset Still’s disease typically presents with high spiking fever, transient rash, polyarthralgia, and generalized lymphadenopathy (25), closely resembling lymphoma’s “B symptoms.” Necrotizing lymphadenitis features sterile nodal necrosis with intense inflammatory infiltration, often presenting with hypermetabolic lymph nodes on PET/CT (26), mimicking lymphoma. Laboratory abnormalities (e.g., elevated ferritin, leukocytosis, CRP) also overlap between these conditions and aggressive lymphoma, diminishing the discriminatory value of individual markers.

SUVmax, although widely used, is notoriously limited: benign inflammatory diseases may exhibit SUVmax levels comparable to lymphoma (6, 27). Our previous study demonstrated that SUVmax was not an independent discriminator, underscoring the unreliability of traditional metabolic parameters. In addition, considerable overlap in lymph node size was observed between benign and malignant conditions in FUO, as benign diseases may also present with confluent lymphadenopathy, while certain lymphoma subtypes can manifest with relatively small nodes. In our study, both metabolic and morphological criteria were applied for node selection, and lymph node size was not retained as an independent predictor in the final model, suggesting that it was not a key determinant of model performance.

In this context, we first constructed a biomarker-based model identifying hyperpyrexia, rash, PCT, and SAA as independent predictors. We then incorporated PET/CT radiomics and utilized LASSO to select key PET and CT texture features that reflect microstructural and metabolic heterogeneity. Finally, we integrated clinical, laboratory, and radiomics predictors to build a comprehensive combined model—achieving superior performance to any single modality.

Radiomics quantifies subtle heterogeneity and texture variations invisible to human observers, reflecting underlying pathophysiology. Its advantages align well with lymphoma’s inherent tumor heterogeneity and the inflammatory patterns of benign disorders. Previous studies have shown that PET/CT radiomics-based machine learning models can achieve promising performance in differentiating lymphoma-related lymphadenopathy in FUO, although most relied solely on imaging-derived features (20). In contrast, the present study incorporates clinical and biomarker data, achieving both enhanced performance and clinical relevance.

Compared with previous studies, the multimodal fusion strategy employed in this work demonstrates clear advantages. Chen et al. (9) analyzed 524 patients with fever of unknown origin (FUO), but their study focused on broad etiological screening rather than specifically differentiating lymphoma from benign lymphadenopathy. Another study (6) proposed a PET/CT plus clinical-parameter scoring system achieving an AUC of 0.93; however, it included a smaller cohort, did not separately analyze laboratory biomarkers, and did not incorporate a combined model. Research by Wang et al. and Wu et al. also supported the utility of PET/CT imaging combined with clinical indicators, but their variable sets were relatively limited and did not include radiomics features (5, 23). However, it should be noted that although the combined model demonstrated superior performance in both cohorts, the differences did not reach statistical significance in the testing set. This lack of statistical significance in the testing cohort may be primarily attributed to the relatively small sample size and the limited statistical power, particularly given the modest differences in AUC values between models. Nevertheless, the combined model consistently achieved the highest AUC across both cohorts, supporting its robustness and potential clinical value.

The innovation of the present study lies in its integration of PET/CT radiomics with laboratory biomarkers. Radiomics quantifies microscopic intra-lesional heterogeneity, while biomarkers such as ALB, PCT, and SAA reflect systemic physiological and inflammatory states. The combination of these micro- and macro-level descriptors provided complementary diagnostic information. Through logistic regression and machine-learning-based feature selection, the composite model achieved superior performance, with an AUC of 0.970—higher than the multimodal approach reported by Chen et al. (9) (AUC 0.88–0.95) and surpassing the radiomics-only random forest model described previously (testing cohort AUC 0.915) (20). Bootstrap resampling further confirmed the robustness of the findings.

Differences in variable selection between studies largely reflect differences in study design. For instance, predictors such as arthralgia, PCT, high fever, ALB, and SAA emerged as independent risk factors in our analysis but were not included in the model by Chen et al. (9), whereas their identified variables (e.g., weight loss, splenomegaly, ESR) did not remain significant in our cohort. Chen et al. stratified FUO patients into infectious, malignant, and inflammatory categories and therefore required broad etiologic indicators, whereas our study focused specifically on distinguishing lymphoma from benign lymphadenopathy, enabling more targeted selection of variables aligned with their distinct pathophysiologic characteristics. For example, high fever is often associated with active lymphoma or early inflammatory responses, and hypoalbuminemia reflects chronic catabolic effects of lymphoma, making these biomarkers particularly relevant.

Despite its strengths, this study has several limitations. First, the single-center retrospective design may introduce selection bias, and the disease spectrum and diagnostic pathways of the institution may limit external generalizability. Second, the sample size was relatively modest, raising the possibility of mild overfitting. Third, radiomics feature extraction relied on manual VOI delineation, which—although performed by two experienced physicians—may still introduce subjective variability; additionally, the study did not include data from different PET/CT vendors, limiting feature standardization. Finally, the biomarker panel focused on conventional inflammatory and clinical indices, without incorporating newer molecular markers such as circulating tumor DNA or microRNAs, which may carry additional diagnostic value. Future studies with larger, multicenter external validation cohorts are warranted to further validate the robustness and generalizability of the combined model.

## 5 Conclusion

The PET/CT radiomics model shows strong potential for differentiating benign from malignant lymphadenopathy in FUO. When combined with multidimensional biomarkers such as PCT and SAA, diagnostic performance is further enhanced. This multimodal approach provides a promising direction for improving the accuracy and clinical applicability of FUO diagnostic strategies.

## Data Availability

The original contributions presented in the study are included in the article/**Supplementary Material**. Further inquiries can be directed to the corresponding author.
